# Postnatal Vitamin D Intake Modulates Hippocampal Learning and Memory in Adult Mice

**DOI:** 10.3389/fnins.2018.00141

**Published:** 2018-04-03

**Authors:** Qiujuan Liang, Chunhui Cai, Dongxia Duan, Xinyu Hu, Wanhao Hua, Peicheng Jiang, Liu Zhang, Jun Xu, Zhengliang Gao

**Affiliations:** ^1^Shanghai Tenth People's Hospital, School of Medicine, Tongji University, Shanghai, China; ^2^Advanced Institute of Translational Medicine, School of Medicine, Tongji University, Shanghai, China; ^3^East Hospital, School of Medicine, Tongji University, Shanghai, China

**Keywords:** vitamin D, postnatal development, hippocampus, learning, memory

## Abstract

Vitamin D (VD) is a neuroactive steroid crucial for brain development, function and homeostasis. Its deficiency is associated with numerous brain conditions. As such, VD and its variants are routinely taken by a broad of groups with/without known VD deficiency. In contrast, the harmful effects of VD overdose have been poorly studied. Similarly, the developmental stage-specific VD deficiency and overdose have been rarely explored. In the present work, we showed that postnatal VD supplementation enhanced the motor function transiently in the young adult, but not in the older one. Postnatal VD intake abnormality did not impact the anxiety and depressive behavior but was detrimental to spatial learning and hippocampus-dependent memory. At the molecular level we failed to observe an obvious and constant change with the neural development and activity-related genes examined. However, disrupted developmental expression dynamics were observed for most of the genes, suggesting that the altered neural development dynamics and therefore aberrant adult plasticity might underlie the functional deficits. Our work highlights the essence of VD homeostasis in neural development and adult brain function. Further studies are needed to determine the short- and long-term effects VD intake status may have on brain development, homeostasis, and diseases.

## Introduction

Vitamin D (VD) is a vital nutrient of pleiotropic effects in many organs and biological processes other than calcium, phosphorus homeostasis, and bone mineralization (Eyles et al., 2013; Li, 2014; Lardner, 2015). In the neural system, VD is emerging as a neuroactive steroid crucial for brain development, functionality, and homeostasis. Vitamin D deficiencies during the development and in the adult were associated with diverse brain disorders such as schizophrenia, autism, depression, Parkinson's, and Alzheimer's diseases (Eyles, 2010; Knekt et al., 2010; McGrath et al., 2010; Balion et al., 2012; Anglin et al., 2013; Annweiler et al., 2013). Vitamin D deprivation during the embryonic development had long lasting impact on the offspring with diminished expression of key neural genes, increased lateral ventricle volume, decreased cortical thickness (Groves et al., 2013), and later altered learning, memory and motor function in the adult (Burne et al., 2004b; Féron et al., 2005; McCann and Ames, 2008; Harms et al., 2012; Eyles et al., 2013; Groves et al., 2013).

Vitamin D deficiency is relatively common in pregnant women, children and older people. Therefore VD and its variants are routinely used for diverse groups with/without known VD deficiency as a simple, safe and inexpensive intervention to alleviate disease burden (Harms et al., 2011). In human Parkinson's disease patients, the placebo had a steady worsening neurological outcomes over time and VD supplementation effectively blocked the worsening effect (Suzuki et al., 2013). With animal models, VD supplementation following a period of deprivation was able to restore cellular activity and function (Zhu et al., 2012; Lardner, 2015).

VD overdose could cause lethargy, memory impairment, confusion, and other clinical symptoms and its toxicity, albeit rare, is mostly related to lactogenic errors or abusive fortification in food (Maji, 2012). But the brain functional impairment caused by VD overdose remains essentially unexplored. The neurochemical and behavioral deficits resulted from VD deficiency and overdose are expected to vary at different developmental and adult stages (O'Loan et al., 2007; Byrne et al., 2013). But studies so far focused on VD treatments beginning either from pregnancy or during adulthood. As such, the safe dose and duration of VD supplementation, and the impact of VD deficiency and overdose remain to be investigated for postnatal brain development (Cui et al., 2015).

The hippocampus is crucial for brain function and homeostasis such as learning, memory, and aging. Hippocampal abnormality is associated with numerous neuropsychiatric and degenerative disorders (DeCarolis and Eisch, 2010), whereby a link between VD and hippocampal homeostasis was suggested (Briones and Darwish, 2012; Zhu et al., 2012; Groves et al., 2016). But to date few have studied the impact of postnatal VD intake abnormality on hippocampal development and function such as learning and memory.

In the present study, we sought to determine potential effects caused by postnatal VD deficiency and overdose in adult life. Our results demonstrate that postnatal VD intake abnormality compromises hippocampal learning and memory in the adults by disrupting the expression dynamics of neural activity genes and hence the neural development. Our work highlights the essence of VD homeostasis in brain development and physiology.

## Materials and methods

### Animals and vitamin D diets

Animal protocols were approved by the Institutional Animal Care and Use Committee of Tongji University (TJMED-013-068) and in accordance with the *Guide for the Care and Use of Laboratory Animals* (National Research Council, 2011). After delivery, female mice (C57BL/6) were randomly divided into 3 groups receiving diets supplemented with three different doses of vitamin D3T (AIN93G rodent diet: Trophic, China; deficiency: 0 IU, standard: 1,000 IU, overdose: 10,000 IU). Upon weaning (at 21 days), male pups continued with the same diet as their mothers. Each group was made up of pups from 3 to 4 litters. Animals were group-housed (5/cage) on a 12-h light/dark cycle, and had free access to water and food. The design of the study is depicted in Figure S1.

### Blood analyses

Mice were deeply anesthetized by an intraperitoneal injection of chloral hydrate (350 mg/kg) and blood samples were collected from the orbital sinus of the pups at postnatal 2, 4, 8, 12, and 24 weeks. The level of 25-hydroxyvitamin D3 in the serum (proportional to the levels of VD in the diets) was determined using ELISA assays (Elabscience, China; Figure S2).The concentration of calcium was kept constant between the groups with calcium fortification in the diets, as determined by QuantiChrom™ calcium assay kit (BioAssay system, USA; Figure S3).

### Behavioral tests

Nine to fifteen male pups per group were subjected to behavioral tests at indicated ages. All animals went through 1-day habituation in the behavioral room. The tests were performed on consecutive days in the following order: open field, light/dark, tail suspension, forced swimming, rotarod, and Morris water maze tests. All tests were finished between 8:00 a.m. and 6:00 p.m. and all apparatus were cleaned with 75% ethanol before and after each testing. Testing sessions were digitally recorded and analyzed using a computerized video tracking system (Ethovision XT, version 7, Noldus Information Technology, the Netherlands).

### Open field test (OF)

The open field test provides simultaneous measures of locomotion, spontaneous exploration and anxiety (Walsh and Cummins, 1976). The apparatus is a polyvinylchloride box (40 × 40 × 40 cm) with a floor evenly divided into nine squares. Mice were placed in the same corner of the test box and each was allowed to move freely for 5 min. A mouse was considered to be into the central area when the center of its body was on it. The total distance traveled and the mean velocity were analyzed to evaluate the locomotion. The number of entries into the central square and the duration of time spent in the central square were used to quantify the anxiety level.

### Rotarod test

The motor coordination skill was evaluated by the rotarod test (Jones and Roberts, 1968). The rotarod is a rotating five-compartment cylinder allowing 5 mice to be tested at a time. The test includes two phases: motor learning and motor coordination. During the learning phase, mice were placed on a rotarod rotating at 5 rpm for 5 min. For the motor coordination phase, mice were placed on a rotarod accelerating from 5 to 40 rpm for up to 5 min. Each animal underwent 3 tests at 60 min intervals. The motor coordination skill was represented by the average time spent on the rod without falling down.

### Light/dark test

The light/dark transition test was developed by Crawley (Crawley and Goodwin, 1980), and was widely used to assess anxiety-like behaviors. The apparatus is a polyvinylchloride box (50 × 30 × 30 cm) with two compartments, the dark one (one third) and the light one (two thirds). The dark side was covered with a dark lid and the light one was open illuminated with a 60 W light bulb positioned 40 cm above the upper edge of the box. Between the two compartments is a doorway (7 × 7 cm). For testing, mice were placed in the same corner in the dark compartment and allowed to move freely between the two chambers for 5 min with the time spent in each chamber and the total number of switching recorded and tracked. A mouse was considered to be in the light zone when the center of its body was in it.

### Tail suspension test (TST)

The tail suspension test was performed to evaluate the depression states. TST procedures were previously described (Steru et al., 1985). Briefly, mice were suspended upside down 50 cm above the floor by adhesive tape placed approximately 1 cm from the tip of the tail. Each trial lasted for 6 min. Mice were considered immobile only when they hung completely passive with total duration of immobility recorded.

### Forced swimming test (FST)

Forced swimming test is commonly employed to measure the depression state (Petit-Demouliere et al., 2005; Can et al., 2012). Briefly, mice were forced to swim in a glass cylinder (height 25 cm, diameter 10 cm) containing 20 cm of water at 25°C, from which the mouse could not escape. Each trial lasted and was tracked by video for 6 min with only the last 4 min of the test analyzed for the total duration of immobility. Mice were considered immobile when they hung in an upright position and made only minimal movements to keep their heads above water.

### Morris water maze (MWM)

The Morris water maze was widely used to study hippocampus-dependent spatial learning and memory. We adopted a previous protocol with some modifications (Vorhees and Williams, 2006). The maze apparatus is an open circular pool (122 cm in diameter) filled with water (22 ± 1°C) made opaque by adding white nontoxic polypropylene pellets. The pool was located in the center of an experimental room with multiple extra-maze visual geometric cues hanging on the wall. The pool was virtually divided into four equal quadrants by two principal axes with tracking software. The end of each line demarcates four cardinal points: North (N), East (E), South (S), and West (W). The test includes two phases of the spatial learning and the reference memory (probe trial). For the spatial learning, a hidden platform (8 cm in diameter) was submerged 1.2 cm beneath the water surface and placed in a fixed location in the center of SE quadrants. Mice were released into the pool from one of the four starting positions (N, W, NE, and SW) and the releasing order was changed pseudorandomly between days. The mice were trained for 12 days with 4 trials per day. A trial was finished when the animal found the escape platform or when 60 s had elapsed. For those that failed to find the hidden platform within 60 s, the experimenters picked them up and placed them on the platform for 15 s at the end of each trial. Mice were dried after each trial.

The most common method to assess reference memory is to administer one probe trial 24 h after the last spatial learning trial. In this study, an additional memory test (probe trial 1) at day 8 was given to determine the rate of memory consolidation. To evaluate the reference memory at the end of the learning phase, the platform was removed and a second probe trial (probe trial 2) at day 13 was given 24 h after the last spatial learning. To evaluate the memory persistence and memory loss, a third probe trial (probe trial 3) was conducted at day 28. For all spatial reference memory, the escape platform was removed from the pool and mice were released from a new starting position not used during the learning phase. All mice were evaluated for their swimming distance and time to find the hidden platform, the time spent in the platform quadrant, the number of times crossing the platform zone and the time spent in the platform zone.

### Hippocampus isolation

Mice were anesthetized by an intraperitoneal injection of chloral hydrate (350 mg/kg) and then perfused transcardially with cold 0.1M PBS for 10 min. Brains were removed and were bisected with left hippocampus isolated for RNA preparation. The right hemispheres were post-fixed in 4% PFA at 4°C for 24 h, cryoprotected in 30% sucrose in 0.1M PBS and then sectioned at 30 μm for HE staining and immunostaining analysis.

### Hematoxylin and eosin stain

Coronal brain sections of 30 μm thickness (1 in every 12 sections) were mounted and stained with the hematoxylin and eosin. HE stained sections were analyzed using the Nikon light microscope.

### Reverse transcription-PCR (RT-PCR) and quantitative PCR (q-PCR)

Total RNA was isolated by the Trizol regent (Invitrogen, USA). qRT-PCR was carried out in triplicates using the SYBR Green PCR master mix (Bio-Rad, USA) with Bio-Rad real time analyzer. The β- actin was used as an internal control. All primer sets were subjected to a dissociation curve analysis and produced single peaks.

### Statistical analysis

All data are shown as the mean ± SEM. Data were analyzed by one-way ANOVA test followed by LSD tests for multiple comparisons, except in the case of the long- term memory test, which was analyzed by the nonparametric Kruskal–Wallis test. Values of *P* ≤ 0.05 were considered significant.

## Results

### Postnatal vitamin D supplementation transiently enhanced motor skill development and maturation in the young adult

**Figure 1:**
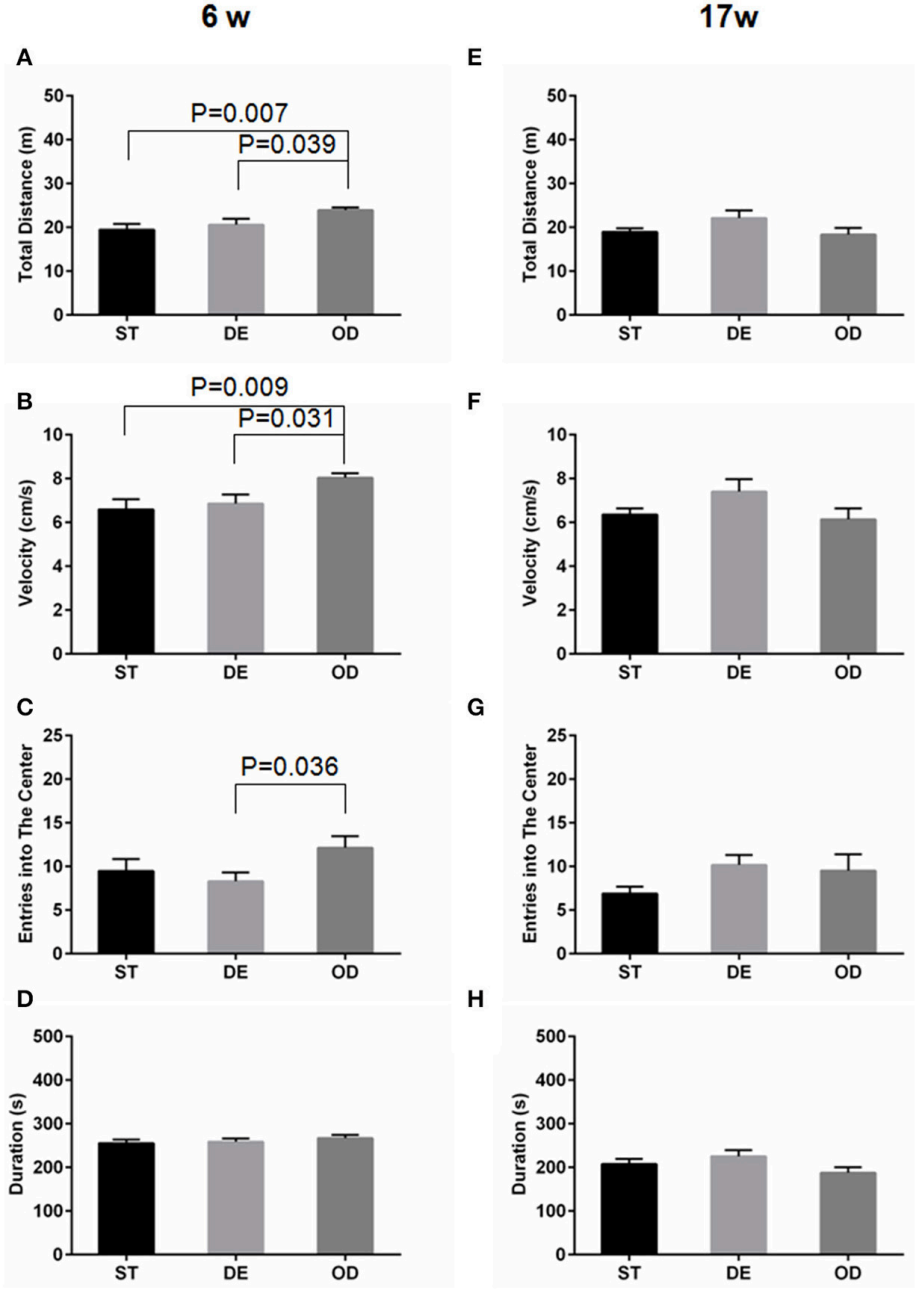
Vitamin D intake status influenced locomotor and exploration activity. Locomotor and exploration activity was examined by open field and rotarod tests for the 6-week **(A–D)** and the 17-week **(E–H)** old mice. **(A,E)** Total distance. **(B,F)** Velocity. **(C,G)** Entries into the center. **(D,H)** Duration on the rod. For the 6-week old mice, but not for the 17-week old ones, the OD group manifested higher mobility and exploration activity. Data are shown as the mean ± SEM. Data were analyzed by one-way ANOVA test followed by LSD tests for multiple comparisons. *n* = 9–15 in each group. ST, standard; DE, deficiency; OD, overdose.

After 6 weeks of VD standard (ST), deficient (DE), and overdose (OD) diets, the OD group traveled more distance and faster than the ST and the DE mice, as determined by “total distance” and “velocity” in the open field test (Figures 1A,B). The OD group had more “entries into the center” when compared to the DE group (Figure 1C). Consistent with an enhanced motor skill, for rotarod test, there was a trend showing that the OD mice spent more time on the rod (duration) than the ST and the DE ones, albeit a significance of *P* ≤ 0.05 was not reached (Figure 1D). However, by week 17, the difference disappeared (Figures 1E–H). Taken together, these results suggest an advantage of enhanced motor skill resulted from postnatal VD fortification during the early development and maturation. But this advantage disappeared as the two other groups grew and caught up over time.

### Vitamin D intake did not have considerable long-term effect on anxiety or depressive-related behavior

**Figure 2:**
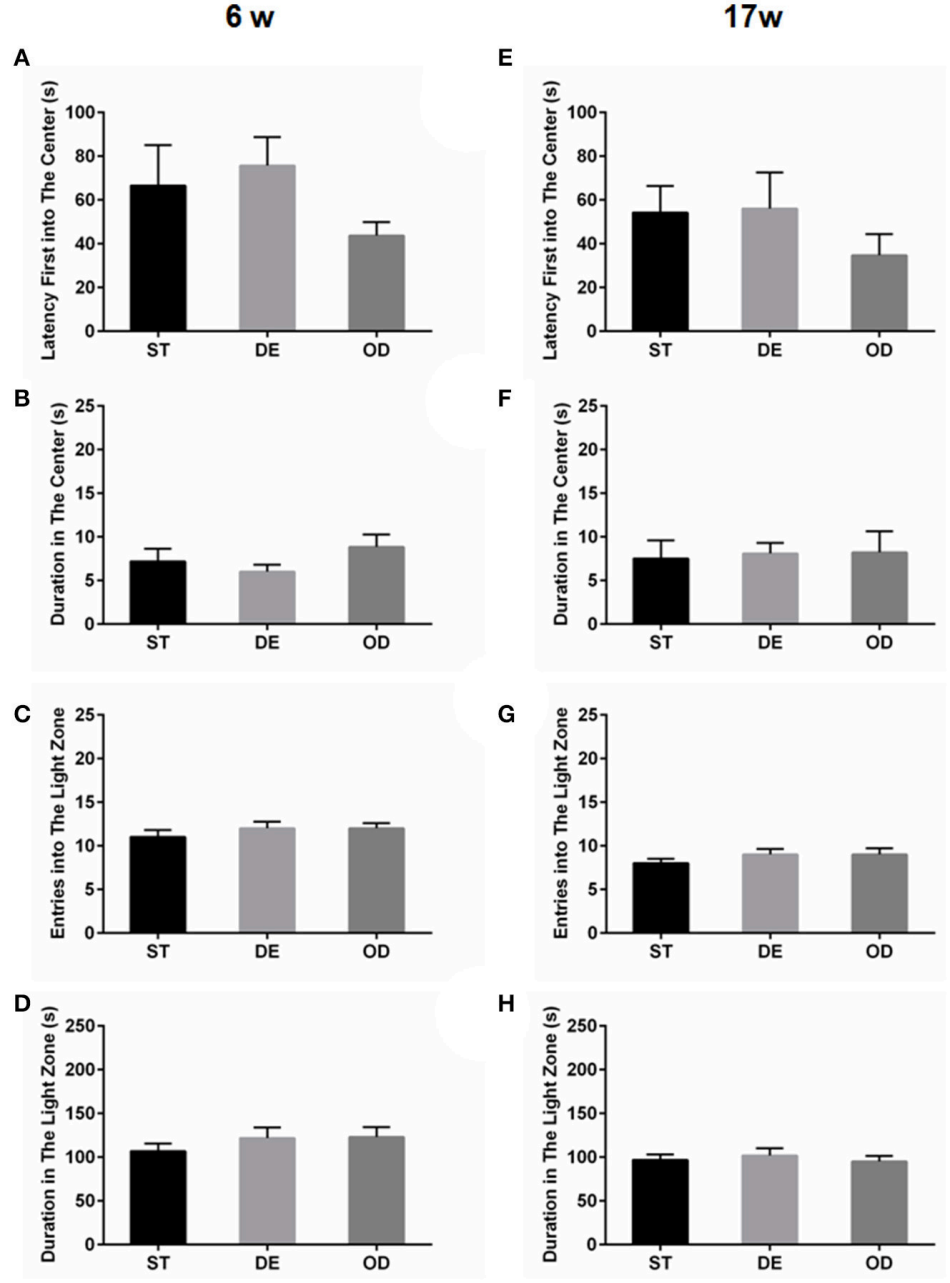
Vitamin D intake did not influence the anxiety related behaviors. The anxiety related behaviors were examined by open field **(A,B,E,F)** and light/dark tests **(C,D,G,H)**, for the 6-week **(A–D)** and the 17-week **(E–H)** old mice. **(A,E)** Latency to the first entry into the center. **(B,F)** Duration in the center. **(C,G)** Entries into the light zone. **(D,H)** Duration in the light zone. Data are shown as the mean ± SEM. Data were analyzed by one-way ANOVA test followed by LSD tests for multiple comparisons. No significant changes were observed between the groups at either time points.

**Figure 3:**
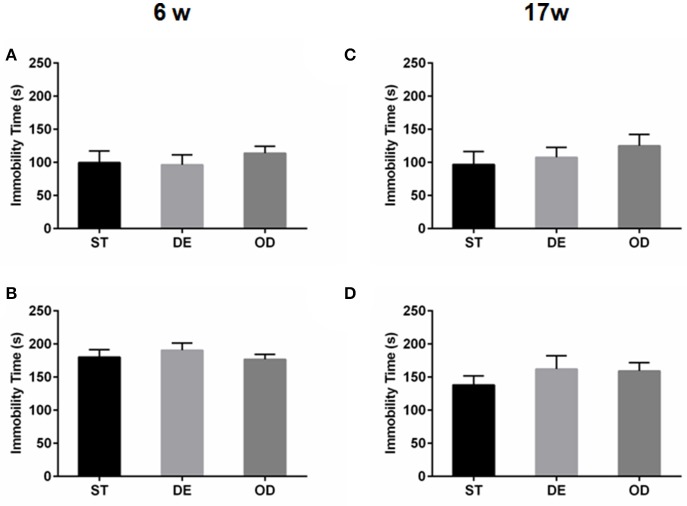
Vitamin D intake did not impact the depressive behaviors. The depressive behavior was determined by tail suspension test **(A,C)** and forced swimming test **(B,D)** for the 6- week **(A,B)** and in the 17-week **(C,D)** old mice. The depression level was illustrated by the total duration of immobility. Data are shown as the mean ± SEM. Data were analyzed by one-way ANOVA test followed by LSD tests for multiple comparisons. There was no difference between the groups at both time points.

The above open field and light/dark tests suggested the anxiety level did not differ between the three groups. No difference was observed in terms of the latency before the first entry into the center (latency first into the center), the duration in the center of open field (Figures 2A,B,E,F), the “entries into the light zone” (Figures 2C,G), and the “duration in the light zone” (Figures 2D,H). Consistent with the above, tail suspension and forced swimming tests did not reveal any significant difference between the groups (Figure 3).

### Vitamin D deprivation interfered with spatial learning ability in mice

**Figure 4:**
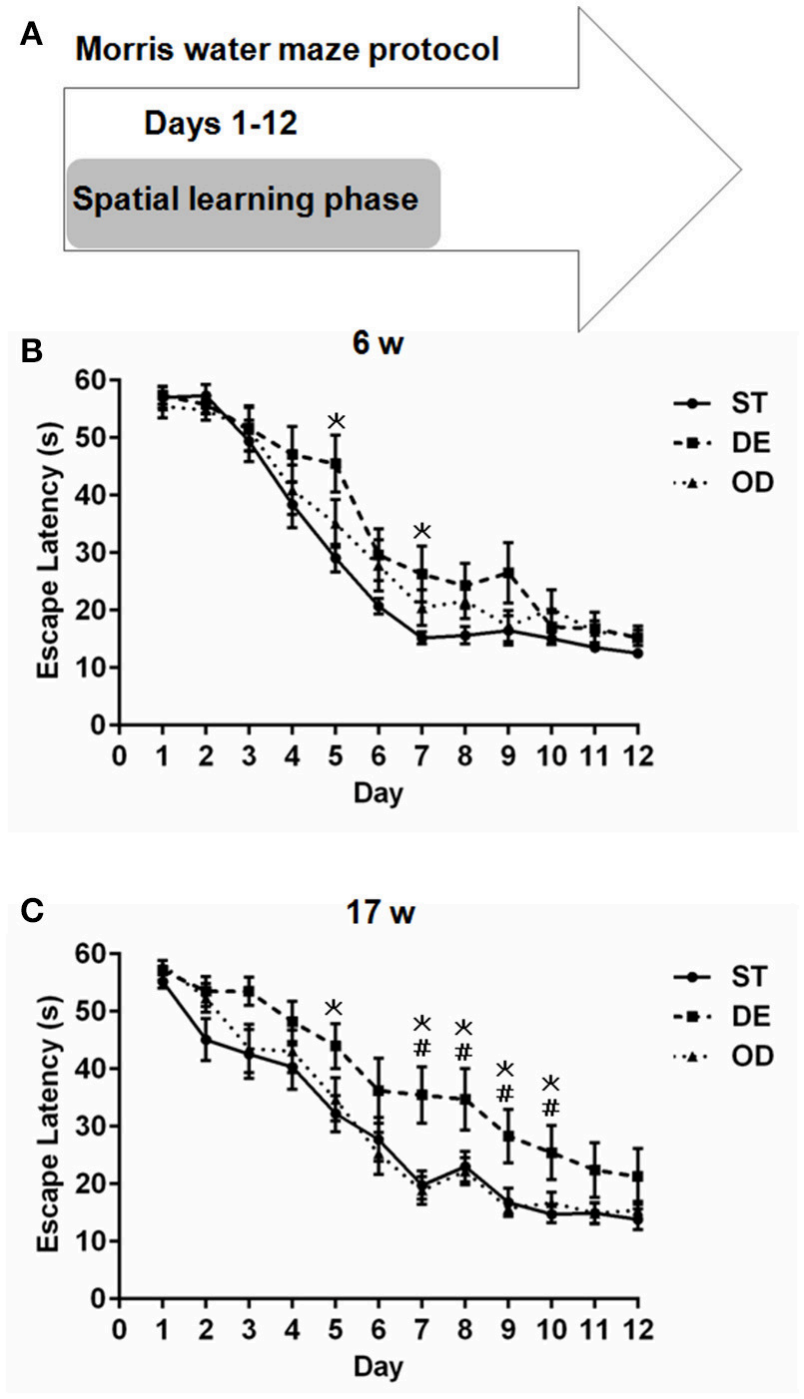
Vitamin D deprivation interfered with spatial learning ability. **(A)** Morris water maze experimental design for the spatial learning phase. Escape latency for the 6-week **(B)** and the 17-week **(C)** old mice during the spatial learning phase. The escape latency for all three groups was progressively reduced with training. For the 6-week old group, we observed a trend that the ST group learned fastest and the DE slowest with the OD in between. The difference between the ST and the DE mice became significant around day 5 (*P* = 0.007) and day 7(*P* = 0.029). For the 17-week old mice, compared to the ST group, the DE was significantly slower on day 5 (*P* = 0.031) and from day 7 to day 10 (*P* = 0.003, 0.031, 0.010, and 0.016 respectively). The difference between the DE and the OD groups was also significant from day 7 to day 10 (*P* = 0.001, 0.018, 0.004, and 0.040 respectively). ^*^represent DE vs. ST: *P* < 0.05; ^#^represent OD vs. DE: *P* < 0.05. Data are shown as the mean ± SEM. Data were analyzed by one-way ANOVA test followed by LSD tests for multiple comparisons. *n* = 9–15 in each group. ST, standard; DE, deficiency; OD, overdose.

Next, we carried out Morris water maze test and determined whether VD modulates hippocampal learning and memory. As shown in Figure 4, for both the 6- and 17-week old mice, the escape latency for all three groups were progressively reduced over the course of 12-day learning period. For the 6-week old subjects, there was a clear trend that the ST group learned fastest and the DE slowest with the OD in between. The difference between the DE group and the ST became significant between day 5 and 7 but further learning eliminates the differences from day 8 to 12 (Figure 4B). For the 17-week old cohorts, the difference between the DE and the OD reached significance between day 7 and 10 but become insignificant with further learning (Figure 4C).

**Figure 5:**
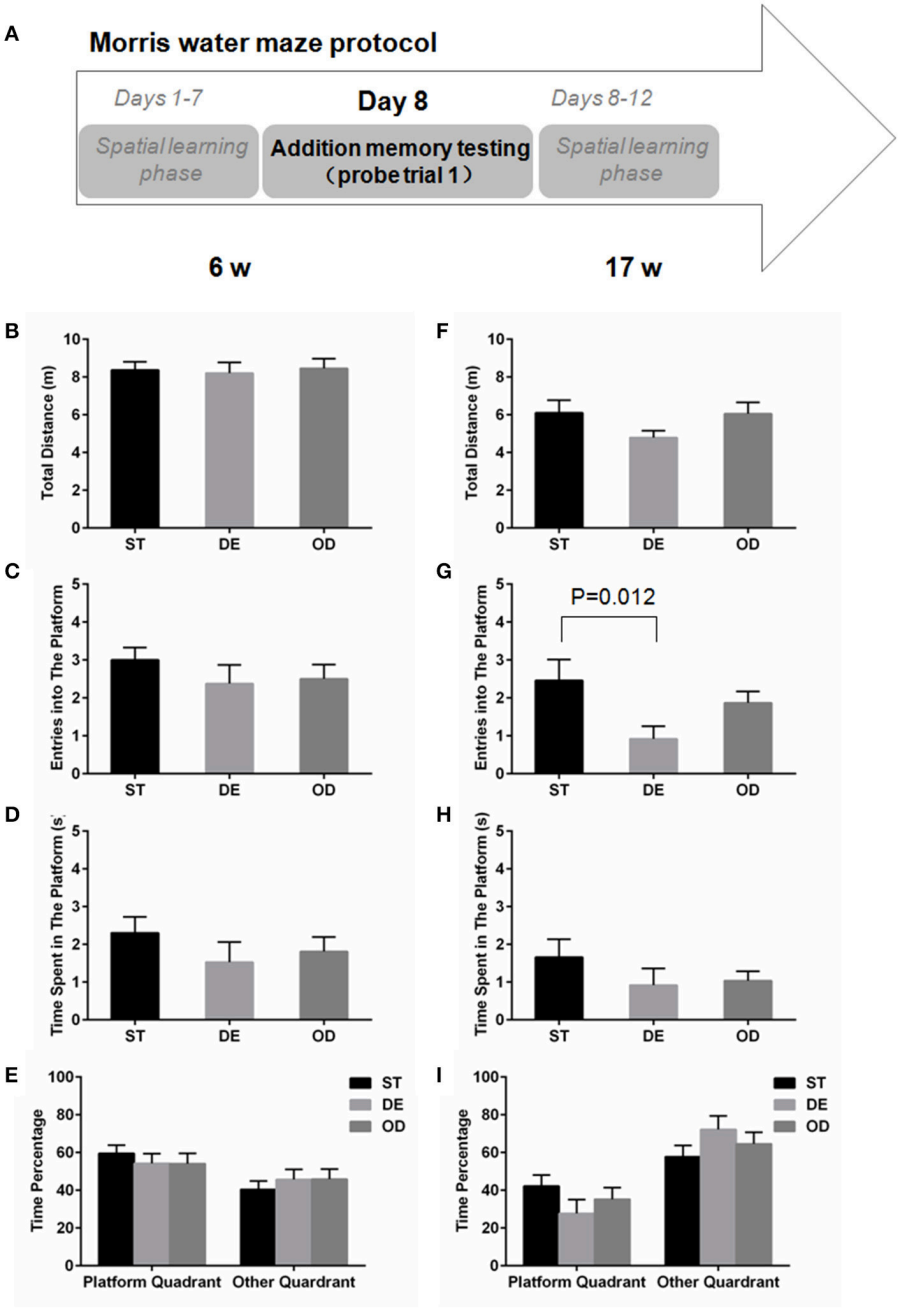
Vitamin D deprivation didn't dramatically interfere with memory consolidation. **(A)** Morris water maze experimental design for additional memory test (probe trial 1). During the spatial learning phase (days 1–12), an additional memory test on day 8 was performed to determine the rate of memory consolidation, for the 6-week **(B–E)** and the 17- week old mice **(F–I)**. **(B,F)** Total distance traveled. **(C,G)** Entries into the platform zone. **(D,H)** Time spent in the platform zone. **(E,I)** the percentage of time in platform quadrant or other quadrant. Data are shown as the mean ± SEM. Data were analyzed by one-way ANOVA test followed by LSD tests for multiple comparisons. Except the entries into the platform zone, for all the parameters analyzed, no significant difference was observed between the three groups.

**Figure 6:**
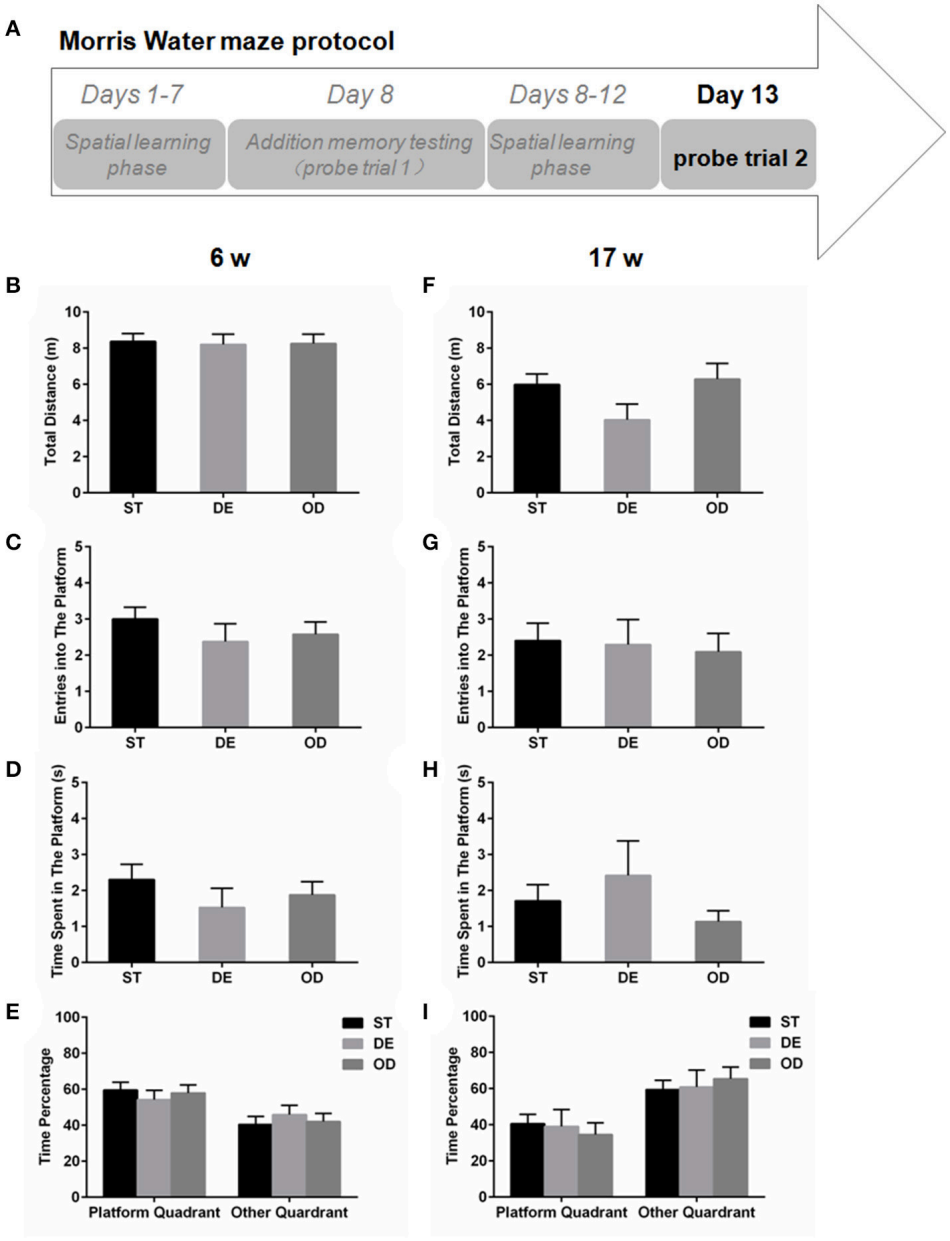
Vitamin D intake didn't alter the spatial reference memory. **(A)** Morris water maze experimental design for the spatial memory test (probe trial 2). The spatial memory test was performed on day 13 for the 6-week **(B–E)** and the 17-week old mice **(F–I)**. **(B,F)** Total distance traveled. **(C,G)** Entries into the platform zone. **(D,H)** Time spent in the platform zone. **(E,I)** The percentage of time in the platform quadrant and the other quadrants. Data are shown as the mean ± SEM. Data were analyzed by one-way ANOVA test followed by LSD tests for multiple comparisons. No significant difference was observed between the groups.

For the 6-week old, neither the additional reference memory test at day 8 (Figures 5A–E) nor the probe trial test at day 13 (Figures 6A–E) produced significant difference with any parameters analyzed. For the 17-week old mice, the additional reference memory test at day 8 (Figures 5F–I) showed that the number of entries into the platform in the DE mice was significantly decreased compared to the ST mice (Figure 5G). But this memory deficit was compensated by further learning and the spatial reference memory test at day 13 did not produce significant difference anymore (Figures 6F–I).

### Abnormal vitamin D intake modulates long-term memory and promoted forgetting

**Figure 7:**
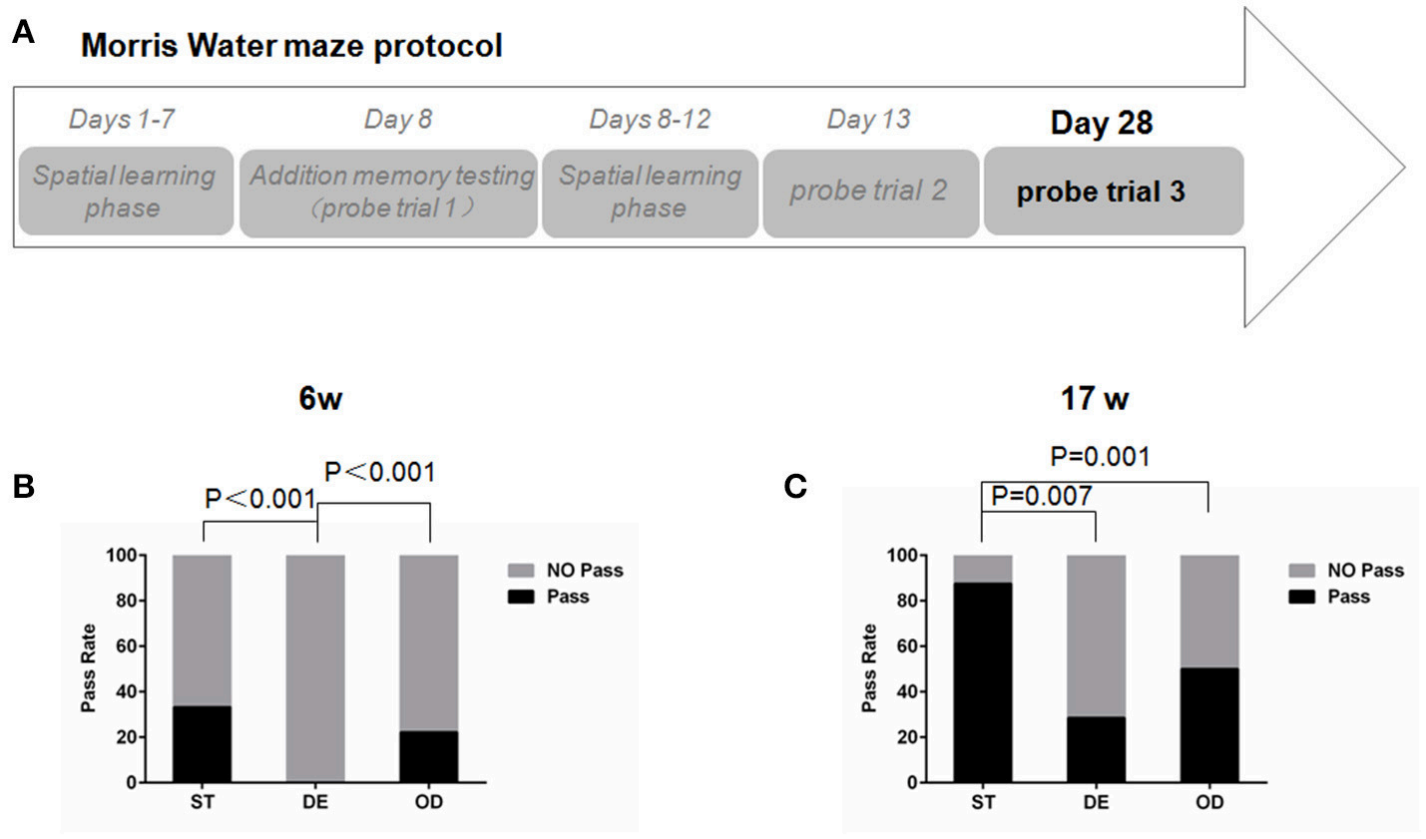
Vitamin D intake interfered with long-term spatial memory and promoted memory loss. **(A)**Morris water maze experimental design for testing the long-term spatial memory. All mice that had a successful memory test at day 13 were subjected to a third probe trial after 15 days, at day 28. **(B,C)** The percentages of mice passing the platform at day 28. For the 6-week old mice, the percentage of the DE group was significantly smaller than that of the ST and OD groups. For the 17- week old mice, the percentages of the OD and the DE groups were significantly smaller than that of the ST one. Data were analyzed by the nonparametric Kruskal–Wallis test. *n* = 9 in each group. ST, standard; DE, deficiency; OD, overdose.

Despite a learning deficit, the DE group eventually reached the same level of memory through further learning and memory consolidation. Taking advantage of this, we wished to examine how their memory might persist over time. All mice that had a successful spatial reference memory test at day 13 were subjected to a third probe trial 15 days after, at day 28 (Figure 7A). For the 6-week old groups, 33% the ST and 22% the OD mice found the platform again but none of the DE could (Figure 7B). For the 17-week old mice, 87% the ST animals remembered the platform. In contrast, only 29% the DE and 50% the OD did (Figure 7C). These results strongly suggest that abnormal VD intake (either overdose or deficiency) compromises long-term memory and promote memory loss.

### Vitamin D intake modulates the expression dynamics of neuronal activity genes in the hippocampus

**Figure 8:**
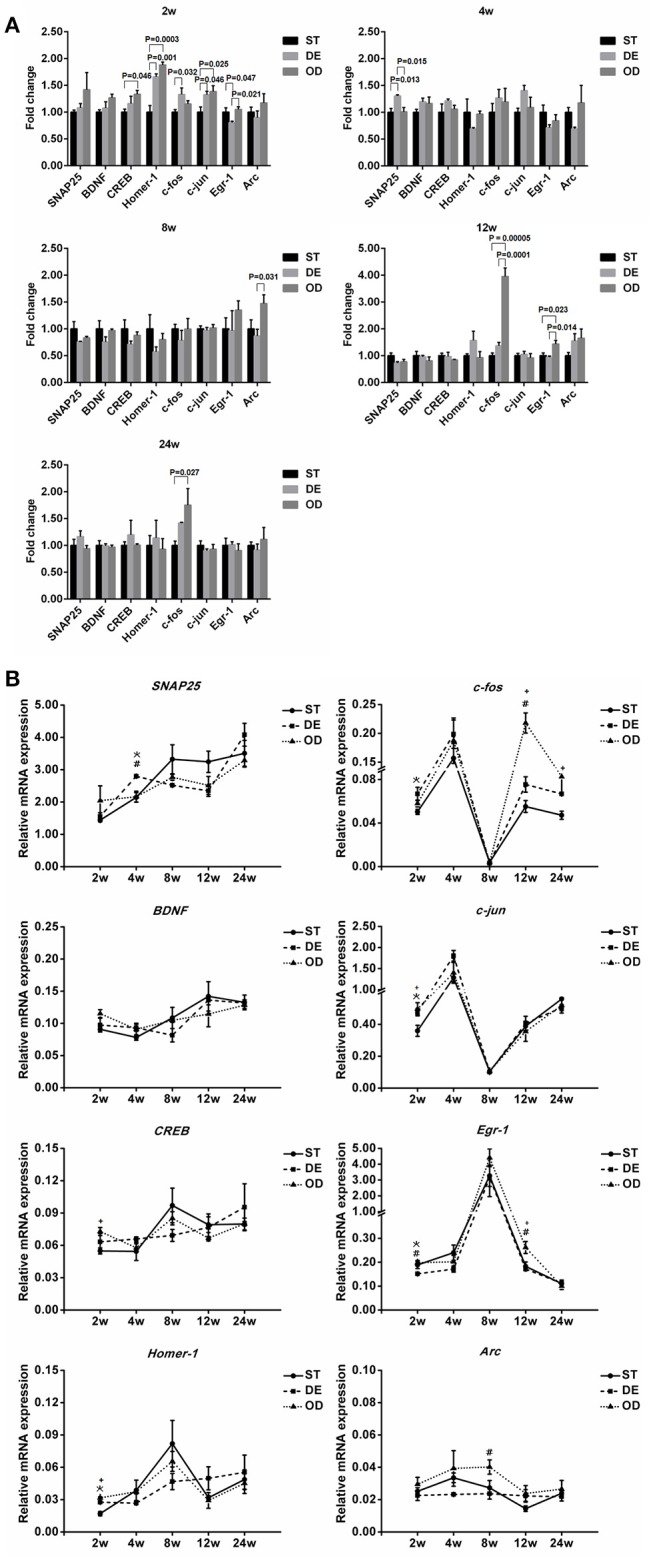
Vitamin D intake modulates the developmental expression dynamics of neuronal activity genes in the hippocampus. The expression of many neuronal activity and memory related genes, including *SNAP25, BDNF, CREB, Homer-1, c-fos, c-jun, Egr-1*, and *Arc*, were examined by qRT-PCR with β-actin as the internal control. **(A)** Relative gene expression was graphed as fold changes relative to the ST control group. Significant changes were observed at various developmental stages for several genes. Compared to the ST mice, the expression of *CREB* was significantly elevated at week 2 in the OD mice while *c-fos* was induced in the DE group at week 2 and in the OD group at week 12 and 24. *SNAP25* was induced in the DE group at week 4 and *Arc* was increased in the OD group at week 8. *Egr-1* was repressed in the DE group at week 2 and induced in the OD group at week 12. Nonetheless, a constant change throughout the developmental and adult stages was not observed for any of the genes examined. ^*^represent DE vs. ST: *P* < 0.05; ^#^represent OD vs. DE: *P* < 0.05; ^+^represent OD vs. ST: *p* < 0.05. **(B)** Gene expression dynamics during hippocampal development. When the developmental dynamics of these genes were examined, an interesting changing trend was evident for most of the genes. For some genes (such as *Arc, CREB, Egr-1, BDNF*, and *Homer-1*), their developmental dynamics were repressed drastically in the DE group and modestly in the OD group. In contrast, for the others (such as *SNAP25, c-jun*, and *c-fos*) their developmental expression became more dynamic in the DE group (to lesser extent in the OD group) than that in the ST groups. Data are shown as the mean ± SEM. Data were analyzed by one-way ANOVA test followed by LSD tests for multiple comparisons. *n* = 3/group.

Consistent with the behavioral observation, expression changes in a number of genes crucial for neuronal activity, learning and memory were readily detectable at various developmental stages. For example, compared to that in the ST mice, the expression of *CREB* was significantly elevated at week 2 in the OD mice while *c-fos* was induced in the DE group at week 2 and in the OD group at week 12 and 24. *SNAP25* was induced in the DE group at week 4 and *Arc* was increased in the OD group at week 8. *Egr-1* was repressed in the DE group at week 2 and induced in the OD group at week 12 (Figure 8A). Nonetheless, a constant change throughout the development and adult stages was not observed for any of the neuronal activity genes examined including *SNAP25, BDNF, CREB, Homer-1, c-fos, c-jun, Egr-1*, and *Arc* (Abel and Lattal, 2001; Messaoudi et al., 2002; Pang and Lu, 2004; Hou et al., 2006; Guo et al., 2010; Gerstein et al., 2013; Kaja et al., 2013; Bannerman et al., 2014; Rivera et al., 2015). When we examined the developmental dynamics of these genes (Figure 8B), an interesting changing trend was evident for most of the genes. For some genes (such as *Arc, CREB, Egr-1, BDNF*, and *Homer-1*), their developmental dynamics were repressed drastically in the DE group and modestly in the OD group. In contrast, for the others (such as *SNAP25, c-jun*, and *c-fos*) their developmental expression became more dynamic in the DE group (to lesser extent in the OD group) than that in the ST mice. Taken together, these results suggest VD intake modulates the developmental dynamics and homeostasis of key neuronal activity and memory genes, presumably responsible for the behavioral changes.

## Discussion

With its expanding roles, a link between VD and brain homeostasis is increasingly evident. However, systematic examinations of whether and how postnatal VD status impacts neural development and adult brain function remain scarce. In the present study, our results demonstrate that postnatal VD deficiency and overdose altered the developmental dynamics of major neural development and activity genes, presumably disrupting normal neural development, and homeostasis. Consequentially, these resulted in compromised hippocampal learning and memory in adult life.

### Postnatal vitamin D status and motor skill development

Our results from open field assays suggest VD supplementation enhances motor skill development in young adults, in line with earlier studies showing that VD supplementation improves muscle strength and physical activity (Girgis et al., 2014; Wyon et al., 2016) and vitamin D receptor (VDR) signaling regulates neuromuscular maintenance and enhances locomotive ability (Sakai et al., 2015). Nonetheless, this early advantage from VD supplementation disappeared over time. By week 17, no significant difference could be detected, suggesting VD supplementation speeds up motor skill development. Nonetheless, VD supplementation did not seem to extend the developmental potential and thus the other two groups gradually catch up as their motor skills developed and matured over time. Earlier studies showed that VD deficient rats and mice exhibited spontaneous hyper-locomotion in a novel environment (Burne et al., 2004a, 2006, 2014; Eyles et al., 2006; Kesby et al., 2006; Harms et al., 2008, 2012; Groves et al., 2013). In the present study, we observed the VD overdose mice had more entries into the central area compared to the VD deficient mice. Nonetheless, the locomotor activity was not significantly different between the VD deficient and the control groups. The discrepancy might result from different experimental paradigms and animal models used. Our current work focused on only postnatal VD status but previous studies involved both embryonic and postnatal VD deficiency. In Harms' study, even with exactly the same treatment regime, VD deficiency caused spontaneous hyper-locomotion in the 129/SvJ but not in the C57BL/6J mice (Harms et al., 2008). Another complicating factor might be that VD may affect the body weight and hence exert negative effects on locomotion activity. But we actually observed that the OD mice were heavier than the DE and the ST animals since week 8 (Figure S4). Besides, we measured serum calcium to exclude potentially detrimental effects from declining bone systems and observed no difference between the groups (Figure S3). Taken together, VD supplementation and deprivation impact locomotor activity, but the severity may vary with the animal strains, growth stages and/or experimental paradigms used.

### Postnatal vitamin D status and mood disorders

A good number of epidemiological studies have linked VD deficiency with human depression. The higher serum 25-hydroxyvitamin D concentrations in general correlated with reduced risk of depression (Brouwer-Brolsma et al., 2015; Fu et al., 2015; Gowda et al., 2015; Han et al., 2015; Jaaskelainen et al., 2015; von Kanel et al., 2015). In support of the above, developmental VD deficient rats and VDR knockout mice displayed high anxiety (Kesby et al., 2006; Pan et al., 2014). Nonetheless, several studies reported that VD supplementation did not alter the anxiety or depression states with C57BL/6 mice, nor influenced cognitive or emotion capacities in healthy young adults (Harms et al., 2008; Dean et al., 2011). We, too, failed to observe a significant difference using the C57BL/6 strain. Thus, under what circumstances and how VD may impact mood remains to be further investigated.

### Postnatal vitamin D status and learning and memory

Numerous population studies have linked the VD status to memory and neurodegenerative diseases yet with conflicting implications (Jorde et al., 2006; McGrath et al., 2007; Evatt et al., 2008; Annweiler et al., 2010, 2015, 2016; Balion et al., 2012; Banerjee et al., 2015). Insights from animal studies were also divided (Becker et al., 2005; Taghizadeh et al., 2013; Latimer et al., 2014). The hippocampus encodes and stores short and working memory. In the current study, we utilized Morris water maze, a spatial reference memory test relevant for hippocampal dependent memory (Vorhees and Williams, 2006; Bartsch et al., 2010; Foster et al., 2012; Buzsáki and Moser, 2013). Postnatal VD deficiency compromised spatial learning but overdose had a milder effect. Nonetheless, after 12 days of learning, all three groups learned the task and the spatial reference memory test at day 13 did not produce significant difference. Importantly postnatal VD deficiency, and to lesser extent, overdose were mostly harmful to long-term memory and promoted memory loss as determined by a third spatial memory test at day 28. Taken together, the proper postnatal VD intake might be required for fine-tuning hippocampal function. The effects of VD intake status may be context- and task-dependent and may not necessarily be observed until the task becomes challenging.

### Postnatal vitamin D status and neural developmental dynamics

In support of the speculation, our efforts so far failed to detect any visible anatomical defects associated with VD intake status (Figure S5), which is also in line with earlier studies reporting no obvious brain defects with VDR or CYP27B1 knockout mice (Li et al., 1997; Panda et al., 2001; Eyles et al., 2003). We reasoned that VD homeostasis might be critical for the proper expression of genes critical for neural development and activity. To our initial surprise, with most of the genes we did not observe a significant and/or constant change (either up or down) throughout the time series, except for modest changes with a few genes at isolated developmental time points. Changes became evident only when we examined the developmental dynamics of these genes. For most of the genes the developmental dynamics with the VD deficient group deviated considerably from that of the control group. The deviation between the overdose and the control group was relatively small, except that for several genes, at isolated time points, for example: *Arc* at week 8 and *c-fos* at week 12, the control and the overdose groups differed considerably. These results were rather consistent with the behavioral results. As discussed earlier, we have seen a more dramatic behavioral difference between the VD deficient group and the other two. The difference between the control and the overdose groups were modest and only became evident when the task was demanding as with long-term memory tests.

Taken together, our results are consistent with the notion that the brain is a plastic organ of high robustness and has the molecular and cellular capacity to buffer insults/stress from changing environments and respond accordingly. When not properly developed as caused by VD deficiency or overdose in the present study, the brain would lose the molecular capacity so that some genes might not respond to environmental changes while the others respond too much, similar to what we have observed.

## Concluding remarks

As suggested by mounting evidence, VD is a neuroactive steroid crucial for brain development, homeostasis and diseases. The unique focus of our present study was to investigate the effects on their later adult life resulted from the postnatal VD deficiency or overdose in new born mice. Our work shows that prolonged postnatal VD deficiency or overdose are detrimental to hippocampal development and physiology at least partially by disrupting the developmental dynamics of genes critical for neural development and activity. Consequentially, these abnormalities result in deficits in various brain functions, most evidently learning and memory. A balanced VD intake during postnatal and juvenile stages is crucial for normal brain development and function later in the life, and confers robustness and plasticity on the brain in the changing environments and/or challenging tasks. Abnormal VD intake, especially deficiency, is detrimental to brain health and function. But the exact underlying mechanisms, at what circumstances and how VD intake modulates brain function, homeostasis and diseases remain to be further investigated.

## Author contributions

QL, CC, and ZG conceived the study and wrote the manuscript. QL, DD, and XH performed experiments, and PJ, LZ assisted with behavior experiments. QL, CC, DD, and WH analyzed the data. ZG, JX, and CC provided the funds and supervised the project. All authors reviewed the manuscript.

### Conflict of interest statement

The authors declare that the research was conducted in the absence of any commercial or financial relationships that could be construed as a potential conflict of interest.

## Abbreviations

FST: forced swimming test
MWM: Morris water maze
OF: open field
TST: tail suspension test
VD: vitamin D
VDR: vitamin D receptor.
