# Supplementary material for: Postnatal Vitamin D Intake Modulates Hippocampal Learning and Memory in Adult Mice
Source: Front Neurosci. 2018 Apr 3;12:141. doi: 10.3389/fnins.2018.00141 (PMC5891641; doi:10.3389/fnins.2018.00141)
Supplement: Supplementary file 1 [file DataSheet1.PDF]

Supplemental Data

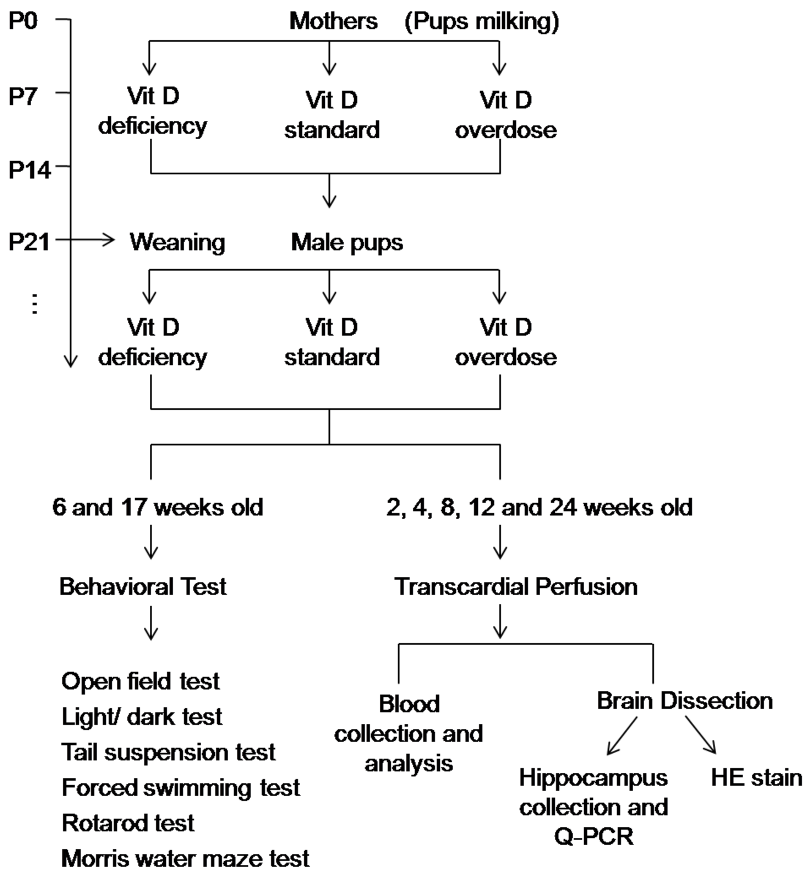

Fig. S1. Overview of the study design.

.

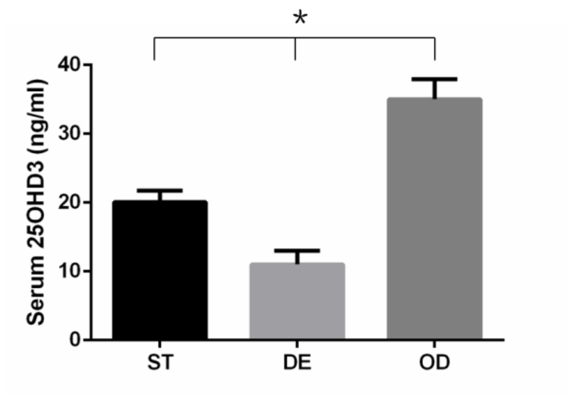

Fig. S2. Serum levels of 25OHD3. Increase in dietary VD resulted in a 2-fold increase in the

serum level of 25OHD3. Data are shown as the mean ± SEM. Data are analyzed by one-way ANOVA test followed by LSD tests for multiple comparisons. n=5 per group. ST: standard, DE: deficiency, OD: overdose. * represent $P < 0.05$.

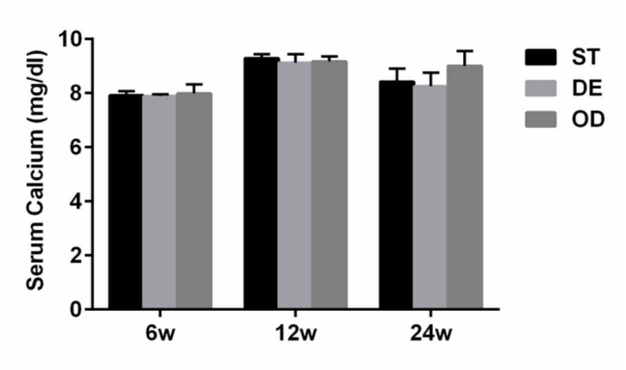

Fig. S3. Serum levels of calcium. The concentration of calcium was kept constant between the groups with calcium fortification in the diets. Data are shown as the mean ± SEM. Data are analyzed by one-way ANOVA test followed by LSD tests for multiple comparisons. n=5 per group in each time point. ST: standard, DE: deficiency, OD: overdose.

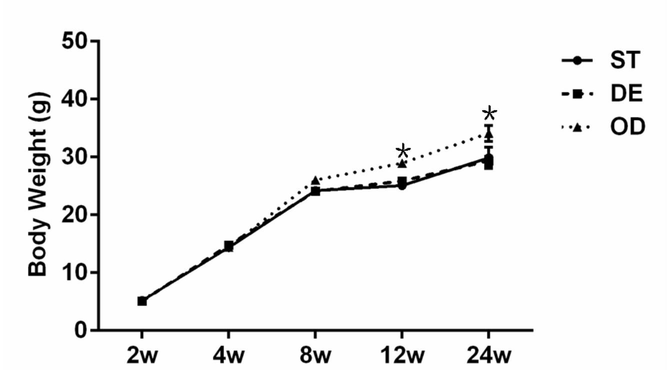

Fig. S4. Vitamin D overdose increased the average body weight of mice over time. The average body weight of the OD group was heavier than that of the DE and ST ones with significant difference observed at week 12 (OD *vs*. ST: $P < 0.0001$; OD *vs.* DE: $P < 0.0001$) and 24 (OD *vs*. ST: P=0.026; OD *vs*. DE: P=0.015). ⭑represent OD *vs*. ST or DE: $P < 0.05$, Data are shown as the mean ± SEM. Data are analyzed by one-way ANOVA test followed by

LSD tests for multiple comparisons. n=5 per group in each time point. ST: standard, DE: deficiency, OD: overdose.

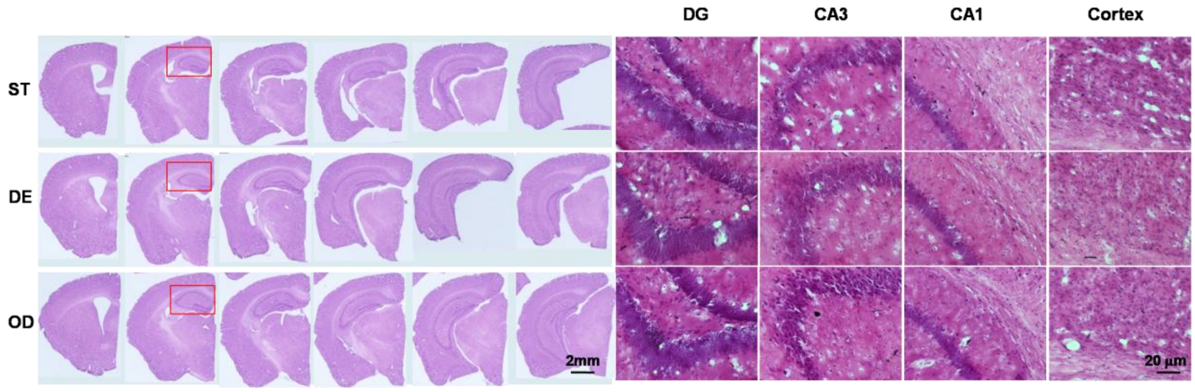

Fig. S5. Abnormal VD intake did not cause visible brain anatomical defects. A series of brain sections from the rostral to the caudal were stained with hematoxylin and eosin. ST: standard, DE: deficiency, OD: overdose, DG: dentategyrus.
